# Evaluation of a New Mobile Virtual Reality Setup to Alter Pain Perception: Pilot Development and Usability Study in Healthy Participants

**DOI:** 10.2196/52340

**Published:** 2024-12-11

**Authors:** Samuel E J Knobel, Raphael Oberson, Jonas Räber, Narayan Schütz, Niklaus Egloff, Angela Botros, Stephan M Gerber, Tobias Nef, Lukas Heydrich

**Affiliations:** 1 Gerontechnology & Rehabilitation Group, University of Bern, Bern, Switzerland; 2 CORE Lab, Psychosomatic Competence Center, Department of Neurology, Inselspital, Bern University Hospital, University of Bern, Freiburgstrasse 41, Bern, 3010, Switzerland, 41 31 632 70 00; 3 Psychosomatic Competence Center, Department of Neurology, Inselspital, Bern University Hospital, University of Bern, Bern, Switzerland; 4 ARTORG Center for Biomedical Engineering Research, University of Bern, Bern, Switzerland

**Keywords:** immersive virtual reality, embodiment, pain management, chronic pain, full-body illusion, cardiovisual illusion, pain, virtual reality, pilot study, development, mobile virtual reality, mobile, virtual environment, usability, heart rate, mobile phone

## Abstract

**Background:**

Chronic pain presents a significant treatment challenge, often leading to frustration for both patients and therapists due to the limitations of traditional methods. Research has shown that synchronous visuo-tactile stimulation, as used in the rubber hand experiment, can induce a sense of ownership over a fake body part and reduces pain perception when ownership of the fake body part is reported. The effect of the rubber hand experiment can be extended to the full body, for example, during the full-body illusion, using both visuo-tactile and cardiovisual signals.

**Objective:**

This study first aimed to evaluate the usability and accuracy of a novel, mobile virtual reality (VR) setup that displays participants’ heartbeats as a flashing silhouette on a virtual avatar, a technique known as the cardiovisual full-body illusion. The second part of the study investigated the effects of synchronous cardiovisual stimulation on pain perception and ownership in 20 healthy participants as compared with asynchronous stimulation (control condition).

**Methods:**

The setup comprised a head-mounted display (HMD) and a heart rate measurement device. A smartphone-based HMD (Samsung Galaxy S8+) was selected for its mobility, and heart rates were measured using smartwatches with photoplethysmography (PPG). The accuracy of 2 smartwatch positions was compared with a 5-point electrocardiogram (ECG) standard in terms of their accuracy (number and percent of missed beats). Each participant underwent two 5-minute sessions of synchronous cardiovisual stimulation and two 5-minute sessions of asynchronous cardiovisual stimulation (total of 4 sessions), followed by pain assessments. Usability, symptoms of cybersickness, and ownership of the virtual body were measured using established questionnaires (System Usability Scale, Simulator Sickness Questionnaire, Ownership Questionnaire). Pain perception was assessed using advanced algometric methods (Algopeg and Somedic algometer).

**Results:**

Results demonstrated high usability scores (mean 4.42, SD 0.56; out of 5), indicating ease of use and acceptance, with minimal side effects (mean 1.18, SD 0.46; out of a possible 4 points on the Simulator Sickness Questionnaire). The PPG device showed high heart rate measurement precision, which improved with optimized filtering and peak detection algorithms. However, compared with previous work, no significant effects on body ownership and pain perception were observed between the synchronous and asynchronous conditions. These findings are discussed in the context of existing literature on VR interventions for chronic pain.

**Conclusions:**

In conclusion, while the new VR setup showed high usability and minimal side effects, it did not significantly affect ownership or pain perception. This highlights the need for further research to refine VR-based interventions for chronic pain management, considering factors like visual realism and perspective.

## Introduction

### Background

Chronic pain is a major global health care problem [1], affecting about 20% of people worldwide. Approximately 40% of all medical visits are due to chronic pain [2], resulting in annual treatment costs upward of US $600 billion annually in the United States alone [3]. In addition, chronic pain is related to decreased well-being, psychological distress, and high levels of psychiatric comorbidity [4]. Treatment of chronic pain is challenging, and pharmacological approaches are often limited by side effects [5]. Regarding the importance of chronic pain as a major health problem, there is an urgent need for noninvasive, well-tolerated, and easy-to-use alternative treatment options. Besides obvious pharmacological and invasive treatments, there are numerous other physical, psychological, or social approaches that traditionally include psychotherapy, physical therapy, massages, or acupuncture [6,7]. Others have used biofeedback, including heart rate variability–based biofeedback in order to treat chronic pain [8,9]. Another new and promising approach may be the use of virtual reality (VR) [10-12] as a method to enable new forms of treatment.

### Multisensory Body Representation and Pain Perception

Based on the ﬁndings of an association between chronic pain and abnormal central representation of the body schema and in pain perception [13], several studies showed an association between the experimental manipulation of the body schema using multisensory inputs and pain reduction in patients with chronic pain [14-16]. The term body schema refers to the brain’s continuous representation of the position and movement of the body parts in space by integrating multisensory body related information (eg, visual, tactile, and motor) in order to create a coherent perception of one’s own body [17]. The probably best-known experiment about the manipulation of the body schema using multisensory stimulation is the so-called rubber hand illusion [18]. In the rubber hand illusion experiment, ownership (eg, the degree to which we identify with a given body part) over a fake hand is induced when the participants see it being touched in synchrony with their own physical hand. It is believed that the rubber hand illusion is based on the faulty cross-modal integration of visual (where the participant sees the touch) and sensory (where the participant feels the touch) information into a coherent body schema [18]. Others have highlighted the importance of sensory suggestibility [19] and the characteristics required for the rubber hand illusion to occur [20]. The principle of the rubber hand illusion was successfully extended to the full-body illusion by Lenggenhager and colleagues [21], using VR in order to display tactile stimulation of the participants’ backs synchronously with the visual stimulation of a virtual body. Similar to the rubber hand illusion [14], a change in the perception of pain in healthy participants, as well as in patients with chronic pain, was found in several studies using the full-body illusion [15,22,23], suggesting that the identification with a virtual body induces an analgesic effect for the physical body. However, the currently proposed setup has a major drawback in that it necessitates a second person to perform visuo-tactile stimulation. So in the context of home-based telerehabilitation, there is the urgent need for a method that induces body ownership without the need of another person’s presence.

Recent studies found a way to bypass this problem by using interoceptive biosignals (eg, heartbeat). They were able to induce the full-body illusion, showing the participants a cardiovisual stimulation of a virtual body in VR, with a silhouette ﬂashing around the virtual body in synchrony to the participant’s heartbeat (so-called cardiovisual full body illusion) while participants were not able to (consciously) relate the flashing of the silhouette to the heartbeat [24,25]. Cardiovisual changes influencing self-identification have been linked to altered neural activity in specific brain regions, including the bilateral operculum and parietal somatosensory cortex [25,26]. Moreover, transient changes in neural responses to heartbeats, known as heartbeat-evoked potentials, have been observed in the posterior cingulate cortex and insula during alterations in bodily self-consciousness induced by the full-body illusion [27].

### Evaluation of a New Mobile VR Setup

We developed a setup based on the cardiovisual full-body illusion that later could be implemented in the treatment of patients with chronic pain affecting the whole body (eg, fibromyalgia), and not just a body part (eg, complex regional pain syndrome and neuropathic pain after spinal cord injury [15,16]).

In this pilot study, our primary goal was to evaluate an easy-to-use, telerehabilitation-friendly, mobile setup, that reduces the setup complexity signiﬁcantly by using a stand-alone head-mounted display (HMD) for the visualization and a smartwatch to detect the heartbeats using photoplethysmography (PPG). We hypothesized that participants report a high level of usability and a low number of side effects, allowing for the setup to be tested in a larger randomized clinical trial.

As a secondary outcome, the level of ownership with the virtual body and pain perception were assessed using a randomized within-subject design. We hypothesized that synchronous cardiovisual stimulation results in higher ownership ratings of the virtual body and lower pain perception as compared with asynchronous cardiovisual stimulation.

## Methods

### Participants

A total of 20 healthy volunteers were recruited at the University of Bern by word of mouth between April 2021 and January 2022. Inclusion criteria were volunteers aged ≥18 years old, those capable of judgment, those willing to participate in the study (by signing the informed consent form), and those able to follow the study protocol. The exclusion criteria of nonclinical participants were based on self-declaration: no presence of psychosis or major depression with suicidal risk; no history of alcohol or drug abuse; no inability to follow the procedures of the study, for example, due to language problems; no inability to wear an HMD; and no signs of arrythmia, such as atrial fibrillation. As the primary focus of this pilot study was to assess the usability of the device, we aimed for 20 participants, as this number has been shown to be sufficient for usability testing [28].

### Ethical Considerations

The study was conducted in accordance with the latest version of the Declaration of Helsinki [26]. The study protocol, including statistical methods, quality control, and data protection, was approved by the Ethical Committee of the canton of Bern, Switzerland (registration number 2019‐01515). All participants were instructed about the study procedure, and an informed consent form was obtained. This resulted in an anonymized dataset, where all identifiable data are archived separately. Study data were collected and managed using REDCap (Research Electronic Data Capture; Vanderbilt University) electronic data capture tools hosted at ARTORG Center for Biomedical Engineering Research [29]. The participants were not financially compensated for taking part in the study.

### Setup

#### Overview

**Figure 1.**
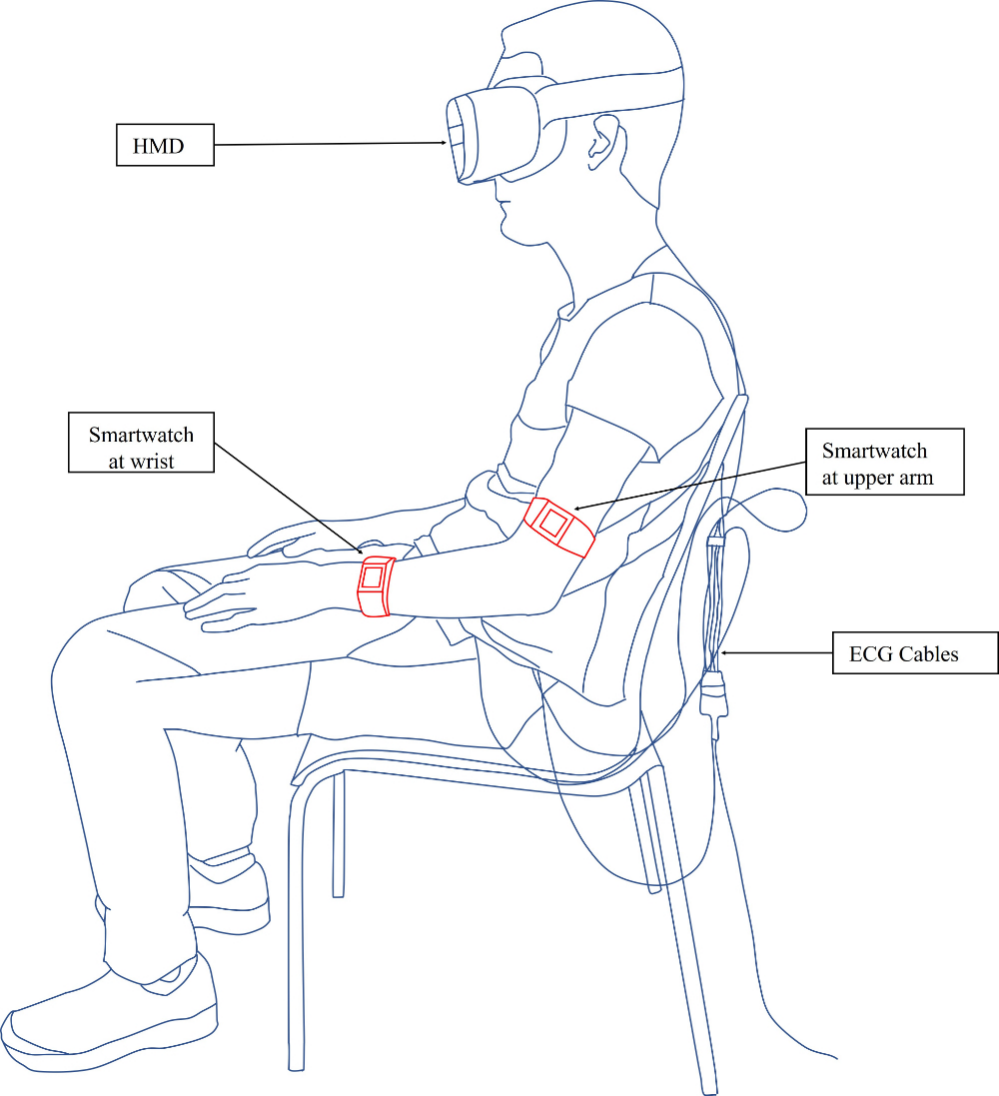
Participant of the study during stimulation wearing an HMD and 2 smartwatches (in red) to measure the heart rate, as well as the electrocardiogram. ECG: electrocardiogram; HMD: head-mounted display.

In Figure 1, the participant is shown with the apparatus. It consists of 2 components: an HMD to visualize the virtual environment and a smartwatch to measure the pulse waves. The 2 devices communicate via the Bluetooth protocol.

#### Head-Mounted Display

The type of used HMD to render the virtual environment is a combination of a smartphone and a holder. The headset itself has two lenses used to focus on the phone screen and a strap to allow ﬁxation on top of the user’s head. Taken together, with the smartphone used as screen, the final resolution is 1480×1440 pixels per eye, the frame rate is 60 Hz, and the ﬁeld of view is 96 degrees. For our experiment, we used the GearVR and a Samsung Galaxy S8+ smartphone.

#### Smartwatch

**Figure 2.**
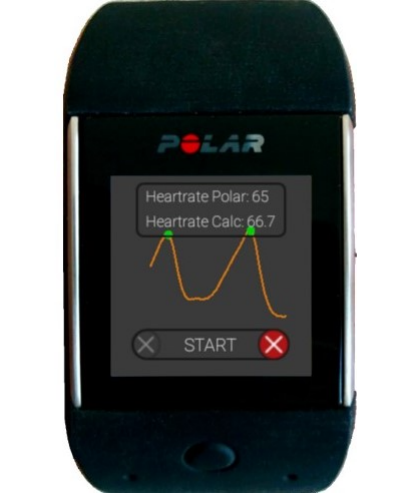
Smartwatch app to start the stimulation, which showed the heart rate suggested by the smartwatch and the calculated heart rate, as well as the pulse wave and where a peak was detected.

The device used is a commercially available smartwatch (Polar M600, Polar Electro Oy), where the raw signal of the pulse wave is measured using PPG at a sampling frequency of 134 Hz. Blood pulse waves are ﬁltered and extracted on the smartwatch (Figure 2). If a pulse wave is detected, the watch sends a signal to the HMD that triggers the appearance of a translucent silhouette around the avatar (synchronous condition). During the asynchronous condition, the translucent silhouette was shown asynchronously to the patients’ heartbeat at a frequency equal to either 80%, 90%, 110%, or 120% of the actual heart rate (shuffling mode). For details of the protocol, see the section “Study Protocol.”

### Virtual Environment

#### Room

**Figure 3.**
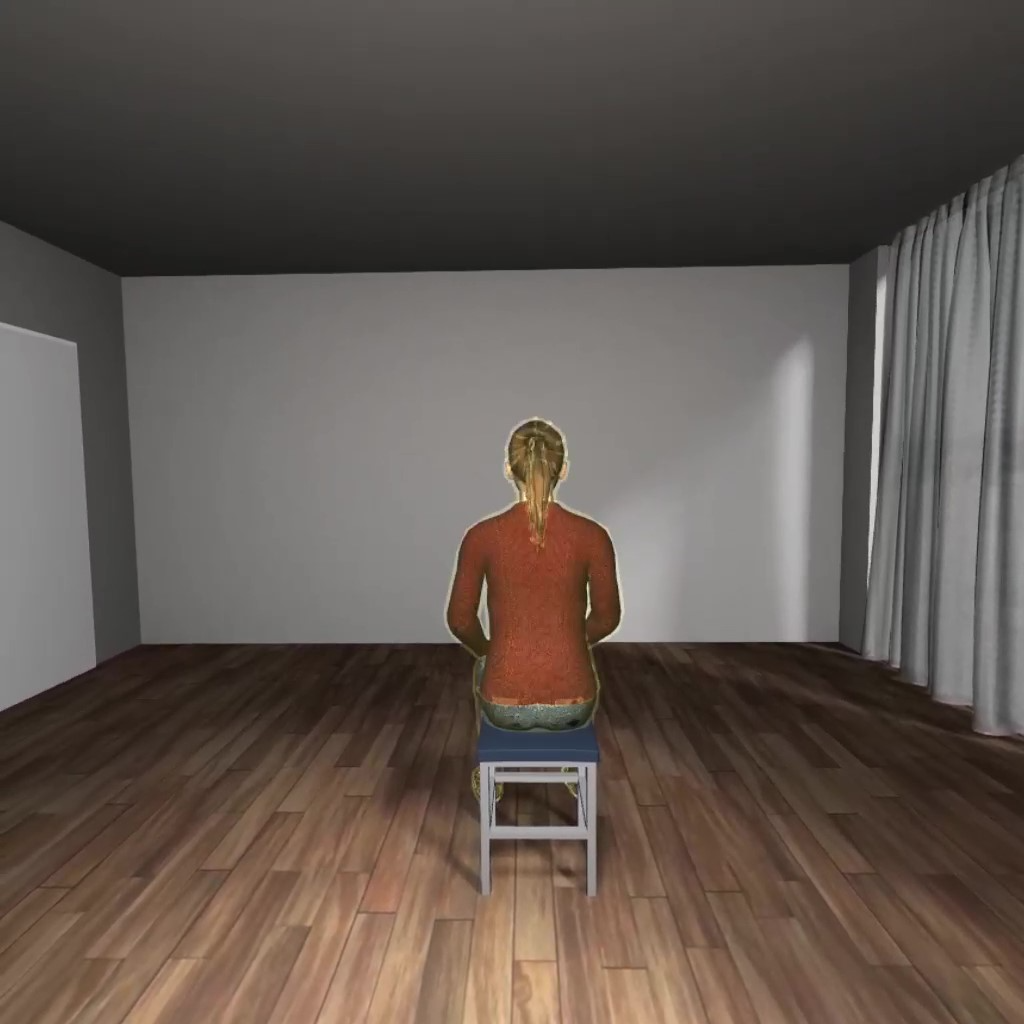
Participants’ frontal view of the virtual environment: room and avatar with the ﬂashing silhouette.

The virtual environment consists of an average-sized (5×6 m), light-ﬂooded room with big, partially curtain-covered windows to see outside. In the room, there is some furniture and a gender-matched avatar sitting on a chair, looking at the wall (Figure 3). The participants are placed in the center, so they see the avatar’s back without being tempted to focus on the furniture or other distractors.

#### Avatar

The 3D avatar is surrounded by a colored and partly translucent silhouette that ﬂashes in synchrony or asynchrony with the heartbeat (see the section “Study Protocol” for details). The ﬂashing is implemented by changing the color of the silhouette between invisible and bright light. When ﬂashing, it immediately changes to the brightest appearance and then slowly fades back to translucent.

### Study Protocol

#### Overview

We used a randomized within-subject design in order to test the level of ownership with the virtual body and pain perception. The study was performed at the ARTORG Center for Biomedical Engineering Research, University of Bern. The test supervisor started the experiment by selecting the gender-matched avatar on the smartphone app. The participants were blinded to the intervention; for example, they did not know that the flashing was synchronous or asynchronous with their heartbeat.

We recorded 2 blocks of 5-minute synchronous stimulation and 2 blocks of 5-minute asynchronous stimulation (total of 4 blocks) in a randomized order and measured pain perception and pain threshold each time before and directly after the intervention (pain intensity [Algopeg and Somedic electronic algometer]). Unrestricted randomization was achieved using Research Randomizer [30] (generated by RO for each participant). Pain perception was always tested first with the Algopeg and then directly with the Somedic algometer (order not changed between blocks). There was a break of 3 minutes between each block.

#### Algopeg

The pressure was applied on the right and left ear lobe and the right and left middle finger, respectively, for 10 seconds each and was invariable. Measurements were done consecutively. The participant was asked to indicate the pain intensity on a numerical rating scale, on which 0 stands for “no pain” and 10 stands for “the most intense pain imaginable.” Since pain subjectively increases during the 10-second stimulation, the participants were explicitly asked about the intensity of pain they perceived at the end of the test (ie, at 10 seconds).

#### Somedic Algometer

The pain pressure detection threshold was measured with a standard electronic algometer by bilateral testing on the middle fingers consecutively (for details, see section on Pain Pressure Detection Threshold). A steadily increasing pressure (50 kPa for 1 s) was applied, thus checking for the threshold at which nonpainful perception of pressure changes to painful perception in response to the gradually increased pressure. The participant had to press a button as soon as the pressure sensation subjectively turned into pain. Thereupon, the algometer froze the value on the display. The procedure was repeated 3 times and the average value was used for data analysis.

Also, ownership of the avatar was assessed for the synchronous and asynchronous condition. At the end of the procedure, acceptance and usability was assessed for the whole experimental setup (see section on ownership). The whole study procedure lasted about 45 minutes. The study methods, protocol and measurements were not changed throughout the study.

### Outcome Measurements

#### Usability

For measuring the acceptance and usability, as well as negatie symptoms, a combination of the System Usability Scale (SUS) [31] and the Simulator Sickness Questionnaire (SSQ) [32] was used. This merged questionnaire was asked once after each condition. The SUS items are based on a 5-point Likert scale, ranging from strongly disagree (1) to strongly agree (5). We used 3 items from the SUS (I thought the system was easy to use; I think that I would like to use this system frequently; I felt very confident using the system). The SSQ items are based on a 4-point Likert scale, ranging from none to slight, moderate, and severe. We used 7 items from the SSQ (general discomfort; stomach awareness; sweating; headache; eye strain; nausea; dizziness).

#### Algorithm Validation

To test the reliability of the algorithm, the PPG signal from the device and from the reference electrocardiogram (ECG) was compared. The ECG system had clinical-grade quality and a sample frequency of 240 Hz. Even though the ECG-signal was not perfectly clean all the time due to minor motion, breathing, or sweating artifacts, we managed to detect beats in the ECG signal and annotated them as ground truth. On both signals, the peak-to-peak intervals and how many beats were missed were analyzed at the same time. The number of missed beats was simply counted visually by checking for gaps between the peak-to-peak intervals from the PPG signal and the reference ECG-signal.

#### Pain Sensitivity

Analgesic effects were assessed by pain sensitivity using the Algopeg and the Somedic electronic algometer [33].

### Algopeg

The algometric measurement method to detect hyperalgesia was carried out by means of a pressure pain provocation test [33]. For this type of algometry, we used a standardized peg with a clamping force of exactly 10 N at an extension of 5 mm (Type Algopeg, size 78×10 mm, polypropylene and nickel, spring reinforced in Switzerland; Annette Kocher, Inselspital Bern). High numerical rating scale values correspond to high pain intensity [34]. The mean values for the right and left ear lobe and the right and left middle finger were calculated and used for statistical analysis.

### Pain Pressure Detection Threshold (Somedic Electronic Algometer)

The pain pressure detection threshold was measured with a standard electronic algometer (Somedic Type II, size 161×170×30 mm, probe of 1 cm^2^; Somedic Production AB). The electronic algometer was calibrated following the standard protocol as recommended by the manufacturer [35] and set to deliver. Low thresholds correspond to high pain sensitivity [34]. The mean value for the right and the left middle finger was calculated.

Positive values refer to an increase of pain sensitivity (Algopeg) but a lower pain threshold (Somedic), respectively. The difference of the pre- and postintervention measurement for synchronous and asynchronous stimulation was calculated and averaged over the two repetitions.

### Ownership for the Virtual Body

We assessed ownership for the virtual body using a 7-item questionnaire adapted from Aspell et al [24]. The participants were be asked to indicate how much they agreed with each item using a 7-point Likert scale ranging from 0 (complete disagreement) to 6 (complete agreement). The questions were, for example, “During the experiment, there were times when…I felt as if the virtual body was my body,” “...it felt as if my real body was drifting toward the front,” and “...it seemed as if the flashing semi-transparent template was my heartbeat.”

### Statistical Analyses

All questionnaire data were tested for normality (Kolmogorov-Smirnov test of normality) and, if normal, were analyzed using a 1-sided (1-tailed) *t* test for dependent means or with the Wilcoxon signed rank test. As the quality metric for the algorithm, the root mean square error between corresponding time intervals was calculated over the length of the recordings and over all participants. The significance level was at *P*<.05. Data were analyzed using RStudio (Posit PBC).

## Results

### Demographics

In total, 20 participants (9 male and 11 female) took part in the study. The age ranged from 22 to 29 years. None of them reported prior VR experience.

All the volunteers assessed for the study were eligible. All participants received the intended treatment and finished the study. The data of all participants were included in the analysis.

### Usability

The results of the SUS were normally distributed and show very high usability and acceptance. The mean value of 4.424 (SD 0.56) was signiﬁcantly higher than the midpoint of the scale (*P*<.001; median 4, IQR 4-5). The results regarding side effects measured using the SSQ show minimal to no adverse effects (mean 1.175, SD 0.46; *P*<.001; median 1, IQR 1-1). For details, please refer to Table 1.

**Table 1.** Items from merged questionnaire (System Usability Scale and Simulator Sickness Questionnaire).

Items^a^	Score, mean (SD)	Score, median (IQR)
(1) I thought the system was easy to use	4.54 (0.52)	5 (4-5)
(2) I think that I would like to use this system frequently	4.36 (0.67)	4 (4-5)
(3) I felt very confident using the system	4.36 (0.5)	4 (4-5)
(4) General discomfort	1 (0)	1 (1-1)
(5) Stomach awareness	1 (0)	1 (1-1)
(6) Sweating	1.45 (0.93)	1 (1-1.5)
(7) Headache	1.09 (0.3)	1 (1-1)
(8) Eye strain	1.27 (0.46)	1 (1-1.5)
(9) Nausea	1 (0)	1 (1-1)
(10) Dizziness	1 (0)	1 (1-1)

a Items 1-3 are from the System Usability Scale, based on a 5-point Likert scale (1=strongly agree; 5=strongly disagree). Items 4-10 are from the Simulator Sickness Questionnaire, based on a 4-point Likert scale (ranging from none to slight, moderate, and severe).

### Algorithm Validation

For the validation of the custom peak detection algorithm, its output, meaning the raw list of heartbeat intervals, was compared with the corresponding RR-peak distances (=time) of the ECG reference. Root mean square error between the measured and reference peak-to-peak intervals over all recordings and all participants resulting in 3.4% (SD 2.9%) for the wristwatch and 2% (SD 1.5%) for the watch at the upper arm. This means that the detection algorithm was off in 3.4% and 2% of the cases, respectively. As a second quality measure, we counted the numbers of missed beats of our custom algorithm compared with the ECG reference. Over all participants, more than 6500 beats were detected. The watch located at the upper arm missed only 1 beat, whereas the watch located at the wrist missed 8.

### Pain Sensitivity

The results from both Algopeg and Somedic were normally distributed.

#### Algopeg

The mean change of the pain intensity rating at the finger before and after the synchronous condition was −0.018 (SD 0.29, see Table 2). The mean change of the pain intensity rating at the finger before and after the asynchronous condition was −0.08 (SD 0.22). The changes in the two conditions were statistically not significant different (*P*=.24) (Table 2).

The mean change of the pain intensity rating at the ear before and after the synchronous condition was 0.13 (SD 0.74). The mean change of the pain intensity rating at the ear before and after the asynchronous condition was −0.01 (SD 0.46). The changes in the two conditions were statistically not significant different (*P*=.25).

**Table 2.** Results from the Algopeg and Somedic algometers.

Measure, location, and condition				Values	*P* value
**Algopeg, mean (SD)**					
	**Finger**				.24
		Synchronous		–0.018 (0.29)	
		Asynchronous		–0.08 (0.22)	
	**Earlobe**				.25
		Synchronous		0.13 (0.74)	
		Asynchronous		–0.01 (0.46)	
**Somedic, mean (SD)**					
	**Finger**				.29
		Synchronous		–5.23 (29)	
		Asynchronous		–9.39 (18)	
**Ownership, median (IQR)**					.58
	Synchronous			0 (0-1.5)	
	Asynchronous			0 (0-1.5)	

#### Somedic

The mean change of the pain threshold detection at the finger before and after the synchronous condition was −5.23 (SD 29). The mean change of the pain intensity rating at the finger before and after the asynchronous condition was −9.39 (SD 18). The changes in the 2 conditions were statistically not significant (*P*=.29).

### Ownership

Ratings for ownership (data were not normally distributed) were both low during the synchronous condition and the asynchronous condition (median in both conditions 0). No significant difference was found between the synchronous and asynchronous conditions (Wilcoxon signed rank test Z=1.007, *P*=.59).

## Discussion

### Principal Findings

Our primary goal was to evaluate the reliability of an easy-to-use and mobile VR setup based on the cardiovisual full-body illusion that later could be implemented in the treatment of patients enduring chronic pain. The evaluation included the investigation of the usability and side effects of the new setup as well as the validation of the reliability of the used algorithm. Second, we wanted to test whether synchronous cardiovisual stimulation results in a systematic change of ownership and pain perception in healthy participants. The main finding of this study was that while the VR setup demonstrated high usability and minimal side effects, it did not significantly affect ownership or pain perception.

Regarding the reliability of the used algorithm, we show that the detection of the heartbeat is very reliable and that no relevant deviation of the interpulse-interval compared with the RR interval of ECG was found. Due to its conﬁguration with the sharp edges of the QRS complex, the beat detection in ECG is rather simple [36]. However, the detection of the peak of the blood pulse wave with the same reliability is particularly complicated when live detection, as in the described setup, is required. Due to the position of the PPG measuring sensor being located further apart from the heart (arm and wrist) and wireless communication being slower, compared with the earlier setup using a stationary ECG [24], an additional delay between measuring the peak and displaying it in the HMD is introduced. The ECG measures electric activity and thereby detects the beat even before it reaches the baroreceptors in the periphery. It thus gains some time that can be used for computational time-consuming signal analysis and processing and still show the heartbeat in synchrony with the pulse wave in the periphery. In the new mobile setup, the blood pulse waves in the periphery are used. Thus, less time is available for processing to keep the synchrony between the felt heartbeat and the displayed avatar reaction. The delay introduced by wireless communication and peak detection is around 50 ms, meaning there is a small and constant lag between the pulse wave and the shown beat in the HMD.

In line with the ﬁrst aim, we found very high usability and no relevant side effects (eg, sickness, oculomotor problems, and disorientation). The results are comparable with the ﬁndings of Gerber and colleagues [37]. The presented results are an important prerequisite for the future use of the implemented VR setup in patients even without the help of an experimenter (eg, by patients with a chronic pain condition). Thus, we evaluated the tool to be a reliable, easy-to-use VR setup for the cardiovisual full-body illusion, showing a high level of usability and good tolerance in healthy participants.

The second goal of our study was to investigate the effect of the cardiovisual full-body illusion on ownership and pain perception. We were not able to demonstrate any effect of synchronous cardiovisual stimulation on ownership of the virtual avatar and pain perception. While the pain pressure detection threshold was measured on a continuous scale, ownership and pain intensity were rated on an ordinal scale (Likert scale and numeric rating scale). It can be speculated that a continuous scale would have been more sensitive in order to assess changes of ownership and pain intensity. However, we note that the assessment of the pain pressure detection threshold on a continuous scale did not yield statistically significant results.

### Comparison to Prior Work

While some studies using visuo-tactile stimulation showed a reduction of pain perception in healthy participants [23] and patients with chronic pain [14,15], others did not find any effect of synchronous visuo-tactile stimulation on pain threshold during the rubber hand illusion [38]. In a recent review, Matamala-Gomez and colleagues [12] pointed out that this might be due to the different setups used in the studies. They propose using immersive VR for embodiment of a virtual body, in order to modulate body representation and change pain perception in healthy and clinical populations. Moreover, a previous study has shown that the effectiveness of bodily illusions for pain reduction depends on the visual perspective, for example, the use of a first-person perspective on the virtual body (not a third-person perspective, as in our setup), in order to elicit an analgesic effect [39]. Thus, the absence of ownership and lack of modulation of pain perception in our study might also be related to the use of the third-person perspective.

Also, Solcà et al [16] found that synchronous cardiovisual stimulation reduced pain ratings and improved motor limb function in patients with a chronic regional pain syndrome but not in healthy participants. Thus, this setup needs to be tested further in patients with chronic pain disorders.

Previous studies investigating the effect of cardiovisual stimulation in healthy participants used a video-based setup [24,25]. While we did not directly compare a video-based setup with the computer-generated VR setup using a generic 3D avatar in this study, we speculate that the computer-based VR setup has significant drawbacks if it comes to visual fidelity and visual realism (eg, whether the virtual body resembles the body of the participant). It has been shown that top-down processes, such as visual identity, might influence the rubber hand illusion [40] and the full-body illusion or related paradigms. This is in line with a previous study comparing a video-generated and computer-generated VR setup [41] where no effect on self-location and illusory experience of ownership of more than one body was found in the latter.

### Limitations

First, the sample size of our study was rather small and only included healthy and young participants. This also did not allow us to perform a subgroup analysis. Therefore, it would be interesting to test an improved setup in a larger group of older patients with chronic pain within a randomized clinical trial.

Second, the lack of visual fidelity and realism in the avatar might have influenced the results. Although previous studies suggested that the realism is less important, we cannot comment on this as we did not test this in our study.

Third, the use of a third-person perspective rather than a first-person perspective may have affected the effectiveness of the bodily illusion. Attempts to mitigate these limitations included optimizing the filtering and peak detection algorithms to improve measurement precision.

Fourth, our study was limited to a laboratory setting, and the results may not directly translate to real-world clinical environments.

Finally, it would have been interesting to measure the perceptual drift, for example, if ownership over a virtual body results in dislocation of the perceived self-location from the physical body to the virtual body [21,24,42]. However, given that our study was not able to induce ownership with the virtual body, we do not think that measuring the perceptual drift would have been meaningful in this pilot study. Also, participants already had to be tested for pain threshold and sensitivity as well as ownership right after the manipulation. Therefore, an additional drift measurement seemed not to be feasible or a priority to us at the conception of the study.

### Conclusions and Future Directions

In conclusion, we were able to evaluate a new mobile VR setup that theoretically would allow for the treatment of patients with chronic pain in a home-based telerehabilitation setting. While the setup shows high usability and reliability and seems to be well tolerated, no significant effect regarding the level of ownership with the virtual avatar and modulation of pain perception in healthy young participants could be demonstrated in this study.

Future research should focus on improving the visual fidelity and realism of the virtual avatar and testing the setup with a first-person perspective. In addition, larger and more diverse samples, including older patients with chronic pain, should be included to better understand the potential of this VR intervention.

## Abbreviations

ECG: electrocardiogram
HMD: head-mounted display
PPG: photoplethysmography
REDCap: Research Electronic Data Capture
SSQ: Simulator Sickness Questionnaire
SUS: System Usability Scale
VR: virtual reality
